# Authors’ Response to Peer Reviews of “Mothers’ Knowledge of and Practices Toward Oral Hygiene of Children Aged 5-9 Years in Bangladesh: Cross-Sectional Study”

**DOI:** 10.2196/70145

**Published:** 2025-02-03

**Authors:** Tahazid Tamannur, Sadhan Kumar Das, Arifatun Nesa, Foijun Nahar, Nadia Nowshin, Tasnim Haque Binty, Shafiul Azam Shakil, Shuvojit Kumar Kundu, Md Abu Bakkar Siddik, Shafkat Mahmud Rafsun, Umme Habiba, Zaki Farhana, Hafiza Sultana, Anton Abdulbasah Kamil, Mohammad Meshbahur Rahman

**Affiliations:** 1 Department of Health Education, National Institute of Preventive and Social Medicine, Dhaka, Bangladesh; 2 Department of Public Health and Hospital Administration, National Institute of Preventive and Social Medicine, Mohakhali, Dhaka, Bangladesh; 3 Directorate General of Health Services, Ministry of Health & Family Welfare, Government of the People's Republic of Bangladesh, Dhaka, Bangladesh; 4 School of the Environment, Nanjing University, Nanjing, China; 5 Dental Speciality Center, Dhaka, Bangladesh; 6 BRAC James P Grant School of Public Health, BRAC University, Dhaka, Bangladesh; 7 Credit Information Bureau, Bangladesh Bank, Dhaka, Bangladesh; 8 Department of Business Administration, Istanbul Gelisim University, Istanbul, Turkey; 9 Department of Biostatistics, National Institute of Preventive and Social Medicine, Dhaka, Bangladesh

**Keywords:** mothers’ knowledge and practices, oral hygiene, child oral health, Bangladesh

*This is the authors’ response to peer-review reports for “Mothers’ Knowledge of and Practices Toward Oral Hygiene of Children Aged 5-9 Years in Bangladesh: Cross-Sectional Study.”*

## Round 1 Review

### Reviewer BZ [1]

*This is an interesting piece of research [2], which highlights mothers’ knowledge and practices regarding their children’s oral health in Dhaka City. However, several issues made the study scientifically questionable. The major issues are as follows. The study included mothers from two hospitals in Dhaka City, but the title of the study does not mention this. The sample selection from the mothers visiting the hospitals might not represent general mothers from the whole of Dhaka. Thus, this study might not be generalizable to all mothers in Dhaka City.*

**Response:** The authors are grateful to the reviewers for critically reviewing our manuscript. We agree with the comments. Respondents of this study were the mothers visiting the tertiary-level hospitals of Dhaka City. Generally, the respondents visiting hospitals belonged to all administrative wards (small regions of Dhaka), and it is convenient to get the mothers with children aged 5‐9 years to interview. That is why we chose tertiary-level hospitals to reach the respondents. However, we revised our manuscript title and omitted “Dhaka” from the title. The new title is “Knowledge and practices towards oral hygiene of children aged 5‐9 years old: a cross-sectional study among mothers visited tertiary level hospitals.”

#### Introduction

*Revise the last paragraph of the Introduction to highlight the study gap in Bangladesh and clearly state the objective of the study. Use the formal word “mother” and avoid the word “moms.”*

**Response:** We appreciate the reviewer for this comment. We revised the Introduction of our study and replaced the word “Moms” with mother.

#### Methods

##### Study Setting and Participants

*Give clear reasoning as to why you selected study participants from the hospitals. The last line is confusing. It is not clear whether the participants filled out the questionnaire on their own or they were interviewed by the enumerators.*

**Response:** We are thankful to the reviewer for this comment. Respondents of this study were the mothers visiting the tertiary-level hospitals of Dhaka City. Generally, the respondents who visited hospitals belonged to all administrative wards (small regions of Dhaka), and it was convenient to get this group of mothers with children aged 5‐9 years to interview. That is why we chose tertiary-level hospitals to reach the respondents. However, we revised our manuscript title and omitted “Dhaka” from the title. We interviewed the respondents, and the sentence was revised in our revised manuscript.

##### Sampling Technique

*Please mention the nonresponse bias for the convenient sampling. Give a short description of the pretesting mentioning the number of samples, period, and location for it.*

**Response:** We are again thankful to the reviewer. While we had a 5% nonresponse rate in our final survey, we found less than 5% (2 of 50 mothers refused to be involved in the study) as the nonresponse rate during pretesting of our study. The description of the pretest has been given in our revised manuscript. In our main survey, the nonresponse rate was 2%.

##### Measurement of Knowledge and Practice Score

*Give the 15 knowledge-related questions and 13 practice-related questions in the supplementary file. Mention if these questions are your own or if you used any valid tools or questions adopted from the relevant previous studies. Give adequate information regarding the scoring system of the variables, mentioning the highest possible aggregated score and examples of two questions (one for knowledge and one for practice).*

**Response:** We again appreciate the reviewer. The knowledge and practice questions have been added to the supplementary file (Supplementary Table S1 and Table S2). Both knowledge and practice questions were adopted from reviewing the literature and revised according to our selection criteria. The summation scoring technique was used in computation, and their descriptive statistics, including percentiles, were observed. Then, both the knowledge and practice scores were classified according to percentile, which is evident in the existing literature (reference added). The range for the knowledge and practice scores was 1-15 and 1-11, respectively. In the main text, the section has been revised accordingly.

##### Statistical Analyses

*The authors mentioned that they used the Mann-Whitney U test and the Kruskal-Wallis test. However, they did not mention the underlying assumptions of the tests. Moreover, the Results section also shows the χ*^*2*^ *test but is not mentioned in the Methods section. Furthermore, the last line of the Results of the abstract shows the Pearson correlation coefficient, but nothing is mentioned in the Methods or Results section of the entire manuscript.*

**Response:** We apologize for the mistake. Necessary assumptions were checked before performing statistical analysis. The Statistical Analysis section has been revised and mentions the *χ*^2^ test and Pearson correlation coefficient. All the necessary corrections raised by the editor and reviewers have been addressed.

### Results

#### Table 1

*It is confusing as the text description of Table 1 and the title of Table 1 are different. It is recommended to use two separate tables: one for socioeconomic variables and another for the frequency distribution of the knowledge level among socioeconomic variables. Mention the knowledge- and practice-related raw scores first and then the cross-tab results. There is a major mistake in the results of Tables 1 and 2. The frequency distribution for educational status, occupation, family type, number of family members, and monthly income in Tables 1 and 2 are the same. However, the P values are different. How is this possible? Please check the results.*

**Response:** Please accept our apology for the error that happened unconsciously. The frequency distribution for educational status, occupation, family type, number of family members, and monthly income in Tables 1 and 2 has been rechecked and revised. In addition, Table 1 has been separated into two tables (Tables 1 and 2) and presented accordingly.

### Discussion

*It is confusing whether the practice was for the children or how a mother takes care of their children’s dental health. Mention the implications of your findings rather than just comparing the findings with previous studies. State the limitation of the study, especially the bias regarding convenient sampling. Provide a section on the public health significance of the study findings in Bangladesh.*

**Response:** We sincerely appreciate the reviewer for these comments. The Discussion of the manuscript has been revised accordingly. The limitations have been revised in the Discussion section.

### Conclusion

*The Conclusion section of the study is poorly written and not focused on the findings of the study. Revise the Conclusion section to highlight your study findings.*

**Response:** Thank you again. The Conclusion of the manuscript has been revised accordingly.

### Reviewer AJ [3]

#### Specific Comments

*There were a lot of grammatical issues and typographical errors. The manuscript needs to be edited for grammar and syntax. It is also obvious that the manuscript was not proofread adequately.*

#### Major Comments

##### Abstract

- *A word is missing in the first sentence. Authors should proofread the manuscript.*
- *Keywords: Dhaka is a more appropriate keyword than Bangladesh.*
- *Under the Results in the abstract, respondents should be referred to as such and not as samples.*

##### Introduction

- *The global prevalence of oral diseases was stated, but authors did not capture the prevalence in the study area/country and so have not shown that oral disease is a problem. Even the global prevalence that was stated was only that of dental caries among the seven conditions that make up oral diseases as stated by the authors.*
- *The objective stated here (last sentence) comes off like the authors are assessing the knowledge and practices of oral hygiene with regard to themselves and not their children as stated in the topic.*

##### Methods

- *Was it permission that was given by the institutional review board or an ethical clearance?*
- *This section is quite disorganized. There is a logical flow expected in this section.*
- *Why was a nonprobability sampling technique (convenient sampling) used for this study? The sampling technique was not explained at all. This will make replicating this study difficult.*
- *I have an issue with the scoring system and the grading. Is there a reference for it? I particularly have an issue with “moderately average.” It is not a standard term.*
- *The exclusion criteria are not the opposite of the inclusion criteria as stated by the authors. Exclusion criteria are those already included in the study but that are ineligible for one reason or the other.*

##### Results

- *In the text above Table 1, authors wrote that most respondents (39.3%) had a monthly family income of “21,000‐40,000 taka per month.” This figure (39.3%) is just over one-third of the respondents and not a majority.*
- *Table 1: What is the meaning of graduation and above? Is it graduated secondary school or graduated college?*
- *“Respectively” should be added at the end of the following sentence. “Out of 400 mothers, more than 90% knew the importance of brushing teeth while 82.3% and 80.8% of them knew the recommended frequency and appropriate time for brushing teeth.”*

##### Discussion

- *The second sentence: the study is not evaluating parent’s knowledge and practices but that of mothers.*
- *Grammatical errors and missing words*

##### Reference List

- *Some of the references were not cited correctly. Authors should adhere to the Vancouver referencing style.*

## Round 2 Review

### Reviewer BZ

*The authors impressively amended the initial version of the manuscript based on the reviewers’ comments. However, several issues remain unaddressed.*

*1. The authors should include the city in the title of the study. You can revise the title to “Knowledge and practices towards oral hygiene of children aged 5‐9 years old: a cross-sectional study among mothers visited tertiary level hospitals in Dhaka, Bangladesh.”*

**Response:** Thanks for this suggestion. We revised the title of the manuscript accordingly as “Knowledge and practices towards oral hygiene of children aged 5‐9 years old: a cross-sectional study among mothers visited tertiary level hospitals in Dhaka, Bangladesh.”

*2. Use the full form when it appears first and then use the abbreviation afterward. For example, “KP” in the abstract.*

**Response:** Thanks again for this suggestion. We revised the title of the manuscript accordingly.

*3. Please mention this statistical test in the Methods section of the abstract. You did not mention the χ*^*2*^ *test and Pearson correlation.*

**Response:** Revised the Methods section of the manuscript accordingly as “Statistical analysis including the *χ*^2^ test and Pearson correlation test were performed. The Mann–Whitney *U* test and Kruskal–Wallis one-way ANOVA test were performed to show average knowledge and practice variations among different socio-demographics groups.”

*4. It is recommended to make the recommendation simple and easy to understand for the readers. Avoid duplication of the same term.*

**Response:** Revised the Recommendation section accordingly.

*5. In the sample size calculation, you used P=.58 and P=.57. Please clarify why you used those prevalences. Cite the relevant study here.*

**Response:** The Sample Size Calculation section has been revised accordingly as “A convenient sampling technique was followed for this study. During literature search, no study was found that assessed knowledge and practice towards children’s oral hygiene among Bangladeshi mothers. But, a very few studies found in other country with similar socio-demography (eg, India). Mohandas et al, 2021 in his study entitled ‘Knowledge and practice of rural mothers on oral hygiene for children’ showed the prevalence of knowledge and practice were 58% and 57% respectively [4]. The sample size was calculated using the below equation.

“n =(z^2 pq)⁄d^2 …………………………………… (1)

“the sample size for the mother’s knowledge when *P*=.58 was

“n=(〖1.96〗^2×0.58 × (1‐0.58))/〖0.05〗^2=375

“Similarly, the sample size for mother’s practice level when *P*=.57 was

“n=(〖1.96〗^2×0.57 × (1‐0.57))/〖0.05〗^2=377

“Therefore, we initially chose a maximum of 377 as the required sample size. Considering a maximum 5% non-response rate (based on pre-testing), we rounded up this figure and selected 400 as the approximate sample size in the study.”

*6. Before the heading for the sociodemographic variables in the Methods section, you mention outcome measures. However, the sociodemographic variables are not your outcome variables according to your objectives. You can remove the term outcome measures from here.*

**Response:** The heading “Outcome measure” has been removed from the revised manuscript.

*7. You mentioned that you used 13 questions for the assessment of practices. Thus, according to your scoring approach, there should be a score of 1-13, but here, it is 1-11.*

**Response:** Thank you again. We revised the error. The change is “The range for knowledge and practice score was 1 to 15, and 1 to 13 respectively.”

*8. Please mention the name of the software and version you used for the statistical analysis.*

**Response:** Thank you again. We added the statistical software name with the version as “All the data management and statistical analyses were carried out through IBM SPSS Statistics 25.0.”

*9. Revise the sentence before Table 1. You can make it two sentences. One for family income and another for occupation.*

**Response:** We revised the sentence accordingly as “Majority of the respondents (39.3%) had the monthly family income of 21000‐40000 ($206.19-$392.73) Taka per month. About 13.3% mothers were involved in any paid worked activities (Table 1).”

*10. There is no chi-square–related data in Table 1. Please remove the footnotes from Table 1.*

**Response:** Removed the errors.

*11. In Figure 1, it is recommended to keep the values to one decimal point for 1a and 1b.*

**Response:** Thank you for this suggestion. We removed Figures 1c and 1d in our revised manuscript.

*12. Please revise the sentence before Table 3 to give a clear meaning.*

**Response:** We revised the sentence accordingly as “The educational status (*P*=.002) and income (*P*=.044) were significantly associated with mothers’ oral hygiene practices (Table 3).”

*13. You can remove the percentage symbol from the value and give it in the vertical axis title.*

**Response:** Removed accordingly.

*14. Please give the correlation results in the main manuscript or as a supplementary table.*

**Response:** The correlation results have been given as the supplementary result. Please see Supplementary Result S6.

*15. The authors overlooked the association of knowledge and practice with income and family size. Please give more details on those two points in the Discussion section.*

**Response:** The variable family income has been addressed in the Discussion. Please see page 17 (before the Strengths and Limitation section). Family income has been discussed briefly in the Principal Findings section.
